# Model predictive control (MPC) applied to a simplified model, magnetic nanoparticle hyperthermia (MNPH) treatment process

**DOI:** 10.1088/2057-1976/ad460a

**Published:** 2024-05-10

**Authors:** Ma’Moun Abu-Ayyad, Yash Sharad Lad, Dario Aguilar, Kiana Karami, Anilchandra Attaluri

**Affiliations:** 1 Department of Mechanical Engineering, School of Science, Engineering, and Technology, The Pennsylvania State University-Harrisburg, Middletown, PA 17057, United States of America; 2 Department of Electrical Engineering, School of Science, Engineering, and Technology, The Pennsylvania State University-Harrisburg, Middletown, PA 17057, United States of America

**Keywords:** magnetic nanoparticles hyperthermia treatment, model predictive control, nonlinear control, PI controller

## Abstract

Magnetic nanoparticle hyperthermia (MNPH) has emerged as a promising cancer treatment that complements conventional ionizing radiation and chemotherapy. MNPH involves injecting iron-oxide nanoparticles into the tumor and exposing it to an alternating magnetic field (AMF). Iron oxide nanoparticles produce heat when exposed to radiofrequency AMF due to hysteresis loss. Minimizing the non-specific heating in human tissues caused by exposure to AMF is crucial. A pulse-width-modulated AMF has been shown to minimize eddy-current heating in superficial tissues. This project developed a control strategy based on a simplified mathematical model in MATLAB SIMULINK^®^ to minimize eddy current heating while maintaining a therapeutic temperature in the tumor. A minimum tumor temperature of 43 [°C] is required for at least 30 [min] for effective hyperthermia, while maintaining the surrounding healthy tissues below 39 [°C]. A model predictive control (MPC) algorithm was used to reach the target temperature within approximately 100 [s]. As a constrained MPC approach, a maximum AMF amplitude of 36 [kA/m] and increment of 5 [kA/m/s] were applied. MPC utilized the AMF amplitude as an input and incorporated the open-loop response of the eddy current heating in its dynamic matrix. A conventional proportional integral (PI) controller was implemented and compared with the MPC performance. The results showed that MPC had a faster response (30 [s]) with minimal overshoot (1.4 [%]) than PI controller (115 [s] and 5.7 [%]) response. In addition, the MPC method performed better than the structured PI controller in its ability to handle constraints and changes in process parameters.

## 1. Introduction

Magnetic nanoparticle hyperthermia (MNPH) was approved by the European Medicines Agency in 2010 for the treatment of recurrent Glioblastoma (GBM) in combination with radiation therapy (RT) [1, 2]. MNPH exploits the minimal attenuation of radio frequency AMF by human tissue to heat magnetic iron-oxide nanoparticles injected into deep-seated tumors. AMF interacts with iron oxide nanoparticles to generate heat, predominantly via magnetic hysteresis loss. The heating ability of iron-oxide nanoparticles was quantified using the specific loss power (SLP). SLP is a measure of the thermal power dissipated per unit mass of iron when exposed to AMF.

AMF interaction with electrically conducting (diamagnetic) human tissue induces Foucault or eddy current heating within the tissue [3-5]. For a successful MNPH treatment, the magnetic hysteresis loss power deposited by the iron oxide nanoparticles should be significantly higher than the heat generated by eddy currents. This can be controlled by adjusting the AMF using temperature feedback to ensure an adequate thermal dose deposition [6, 7].

MNPH is used with a pulse-width modulated power input to reduce the inherent effect of eddy heating current [8-10]. Previous work on MNPH control has been demonstrated to improve the heating treatment, reaching hyperthermia temperatures of 43 [°C] to 45 [°C] quickly and precisely [11]. However, a major limitation of the eddy current effect is that it is often overlooked or considered separately from the heating process. In addition, only a few temperature models have been developed for the controller design.

Classical proportional integral derivative (PID) controllers have been used to control temperature because of their simplicity and efficiency trade-off. Du *et al* [12] developed a fuzzy PID controller to compensate for parameter variations during the heating process of MNPH treatment. However, more complex control systems are required for sensitive processes in which very strict tracking is required, such as maintaining hyperthermia in biological tissues. In addition, conventional PID control approaches are limited to controlling high-degree nonlinear systems [13].

MPC has been widely applied in industry and studied in academia [14, 15]. MPC algorithm has gained increasing attention over the last decade owing to improvements in modeling techniques and digital computers [16]. In [17], the authors distinguished between the cost function and the performance metric. The controller was active throughout the entire experiment, but the performance metric was only evaluated in the steady state (i.e., after 400 s). Arora *et al* [18, 19] justified the application of MPC by predicting the exact thermal dosage using a linear thermal dose approximation of Pennes’ bioheat equation [20] without the spatial heat diffusion term. The cost function used in [18] involves the tracking error and plant input, which is very common in MPC applications. Arora *et al* also controlled the thermal dose by adding temperature constraints throughout the study, despite plant identification mismatches. However, the constraints on the tumor and healthy tissue temperatures were independently simulated.

Deenen *et al* [21] highlighted the importance of MPC in hyperthermia to reduce the treatment time, predict future temperature changes due to heat diffusion, and account for the physical constraints of the actuators. Additionally, the accuracy of the required model is depicted as a major challenge because it involves biological tissue parameters that vary spatially, temporally, and with temperature. A further study was conducted by Wordragen [22], who studied two-dimensional MPC for a rotating heating element. Huibregtse *et al* [23] developed a condensed model of temperature dynamics with parameters derived from a simulation of the Pennes’ bioheat equation. In this study, MPC was designed and implemented in an anthropomorphic phantom [23]. Throughout the literature on MPC for MNPH, there has been a focus on achieving precise temperature control in the region of interest (ROI) using models derived from Pennes’ bioheat equation [18, 19]. However, there is no study that accounts for the eddy current heating along with the temperature variations in the ROI.

The present work mainly addresses the non-specific power deposition due to eddy currents to be simulated along with temperature changes in the ROI and the use of AMF amplitude as the manipulated variable. The latter is important because it is directly related to the current passing through the induction heating device, making it easier to control than the power delivered by the equipment. A linear MPC controller was designed to maintain the temperature within the specified threshold value of the nonlinear plant model. Additionally, the MPC was modified to include the time- and temperature-varying parameters of the model within the control action calculation. After this analysis, a simulation of the MPC using a linear model showed that the unconstrained MPC design was insufficient to meet the design criteria. A constrained MPC was proposed and tested over linear MNPH and nonlinear models with the temperature change due to eddy current heating as a constraint. In addition, the constraint on the rate of change in plant input was considered.

## 2. Mathematical modeling of MNPH process

Pennes’ bioheat equation was derived from the three-dimensional Fourier heat diffusion equation, which can model heat distribution in human tissue [24]. Pennes’ bioheat equation [20] presents the temperature rate of change on the left-hand side of equation (1), whereas the spatial heat diffusion, temperature changes, and volumetric heat rate are shown on the right-hand side of the equation (1).


(1) $$\rho_{n}C_{n}\frac{\partial T_{n}}{\partial t}=\nabla \;\;(k_{n}\;\nabla \;T_{n})+\rho_{b}C_{b}\omega_{b,n}(T_{b}-T_{n})+Q_{m,n}+Q_{\text{eddy}}+Q_{\text{MNP}}$$

**Figure 1.**
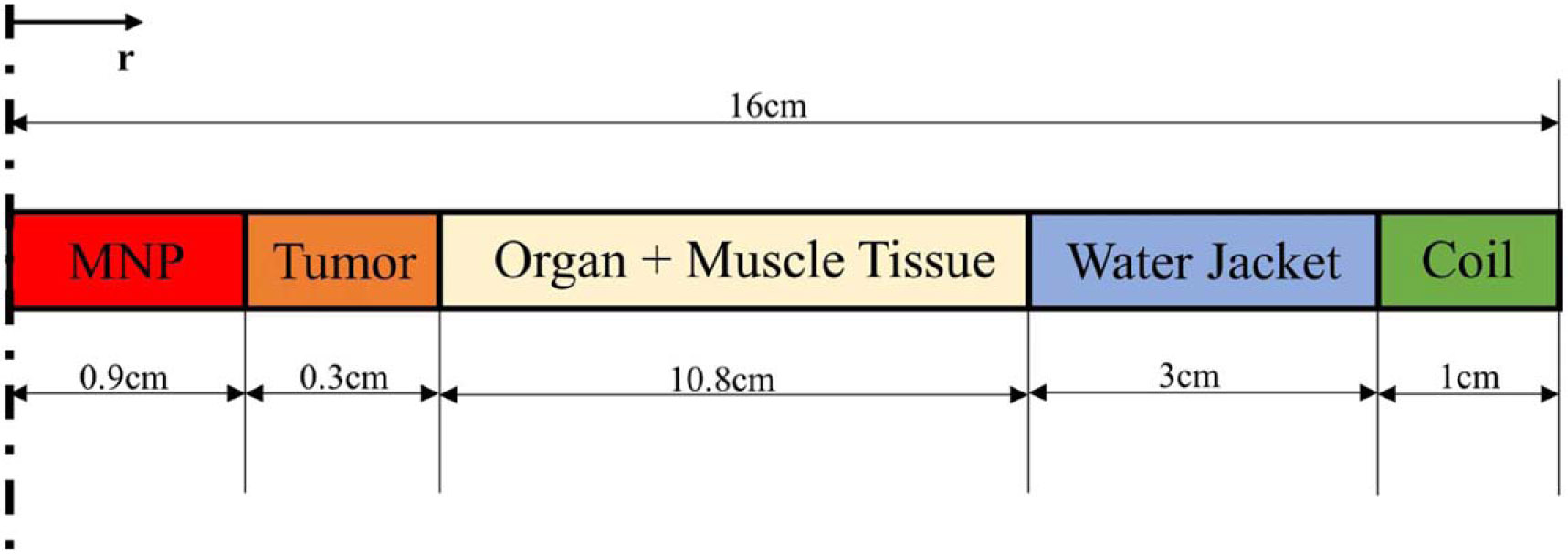
Geometric representation of cylindrical tissue layers.

In equation (1), the subscript n denotes the corresponding layer of the tissue considered and b denotes blood. $T_{n}$ denotes the spatial temperature [K] and $T_{b}$ denotes the blood temperature [K]; ρ denotes the density [kg/m^3^], C is the specific heat [J/kg-K], k is the thermal conductivity [W/m-K], $\omega_{b,n}$ is the perfusion rate in a layer due to blood circulation [1/s], $Q_{m,n}$ is the metabolic heat source [W/m^3^], $Q_{\text{eddy}}$ is the volume power density due to eddy currents, and $Q_{\text{MNP}}$ is the volume power density due to Magnetic Nanoparticles (MNP). Figure 1 shows the geometry of the heat distribution in a single layer of one-dimensional space.

The amount of power transferred from the center of the tumor to the boundary of the tumor can be stated by Fourier’s heat conduction law in one-dimension [25], as described in equation (2).


(2) $$Q_{V}=k_{n}\frac{A}{V}\frac{\text{dT}}{\text{dr}}$$

Where, A is the area of the heat source [m^2^] and V is the volume of the body [m^3^]. It can be seen from figure 1 that the distance between the far side of the tumor and the far side of the muscle tissue is 10.8 [cm]. The heating is induced in the far side of the muscle tissue and due to higher blood perfusion in the muscle tissue, the volume power density Q_v_ which is estimated as 3.6 × 10^3^ [W/m^3^], is insignificant compared to the power applied due to MNP and eddy currents, letting us neglect the heat diffusion term $\nabla .(k_{n}\;\nabla \;\;T_{b,n})$ in equation (1). This helps to reduce the complexity of the model and obtain reduced order model with accurate approximation of state-space matrices. The linear plant models of the tumor and healthy tissue temperatures are defined in equations (3) and (4), respectively, where subscript 1 represents tumor and 2 represents healthy tissue.


(3) $$\rho_{1}C_{1}\frac{\partial T_{b,1}}{\partial t}=-\rho_{b}C_{b}\omega_{b,1}(T_{b}-T_{1})+Q_{m,1}+Q_{\text{lin}1}$$

(4) $$\rho_{2}C_{2}\frac{\partial T_{b,2}}{\partial t}=-\rho_{b}C_{b}\omega_{b,2}(T_{b}-T_{2})+Q_{m,2}+Q_{\text{lin}2}-Q_{\text{free}}$$

(5) $$Q_{\text{lin}1}=1\times 10^{5}(0.5479H-7.8626)$$

(6) $$Q_{\text{lin}2}=1\times 10^{5}(0.1102H-1.2279)$$

In the above equations, $T_{b}$ is the blood temperature, $T_{1}$ is the tumor temperature and $T_{2}$ is the temperature of healthy tissue. $T_{b,1}$ is the difference between blood temperature and the tumor temperature, and $T_{b,2}$ is the difference between blood temperature and the temperature of healthy tissue. $Q_{\text{lin}1}$ and $Q_{\text{lin}2}$ are the linearized input powers to the tumor and healthy tissues, respectively, and H is the amplitude of alternating magnetic field [kA/m]. The linear expressions of these two power values as functions of H are given in equations (5) and (6) [26]. A generalized equation representing inputs and outputs can be formulated as follows.


(7) $$\rho_{n}C_{n}\frac{{\text{dT}}_{b,n}}{\text{dt}}=-\rho_{b}C_{b}\omega_{b,n}(T_{b}-T_{n})+Q_{m,n}+Q_{\text{eddy}}+Q_{\text{MNP}}$$

(8) $$\frac{{\text{dT}}_{b,n}}{\text{dt}}=-\frac{\rho_{b}C_{b}}{\rho_{n}C_{n}}\omega_{b,n}(T_{b}-T_{n})+\frac{1}{\rho_{n}C_{n}}(Q_{m,n}+Q_{\text{eddy}}+Q_{\text{MNP}})$$

(9) $$\left[\frac{{\text{dT}}_{b,n}}{\text{dt}}\right]=\left[-\frac{\rho_{b}C_{b}}{\rho_{n}C_{n}}\omega_{b,n}\right][(T_{b}-T_{n})]+\frac{1}{\rho_{n}C_{n}}[1\;\;1\;\;1]\left[\begin{array}{c} Q_{m,n}\\ Q_{\text{eddy}}\\ Q_{\text{MNP}} \end{array}\right]$$

(10) $$[T_{\text{out}}]=[1][(T_{b}-T_{n})]+[0\;\;0\;\;0]\left[\begin{array}{c} Q_{m,n}\\ Q_{\text{eddy}}\\ Q_{\text{MNP}} \end{array}\right]$$

Where, $T_{b,n}$ is the difference between blood temperature and the temperature of the specific domain into consideration (i.e., tumor or tissue). Since there is no disturbance in the state-space model, the disturbance vector is . Equation (11) gives the volume power density due to free convection cooling where $h_{\text{free}}$ is the convective heat transfer coefficient [W/m^2^-K], r is the radial distance from the center of the body [m], Tb is the blood temperature [K] and $T_{\infty }$ is the temperature at the water jacket [K]. The plant model considering the healthy tissues is shown in equations (12) and (13):

(11) $$Q_{\text{free}}=\frac{h_{\text{free}}}{r}(T_{b}-T_{\infty })$$

(12) $$\left[\frac{{\text{dT}}_{b,n}}{\text{dt}}\right]=\left[-\frac{\rho_{b}C_{b}}{\rho_{n}C_{n}}\omega_{b,n}-\frac{h_{\text{free}}}{r\rho_{n}C_{n}}\right][(T_{b}-T_{n})]+\frac{1}{\rho_{n}C_{n}}[1\;\;1\;\;1\;\;-1]\left[\begin{array}{c} Q_{m,n}\\ Q_{\text{eddy}}\\ Q_{\text{MNP}}\\ Q_{\text{free}} \end{array}\right]$$

(13) $$[T_{\text{out}}]=[1][(T_{b}-T_{n})]+[0\;\;0\;\;0\;\;0]\left[\begin{array}{c} Q_{m,n}\\ Q_{\text{eddy}}\\ Q_{\text{MNP}}\\ Q_{\text{free}} \end{array}\right]$$

where h_free_ is the free convection heat transfer coefficient [W/ (m^2^.K)], Qf_ree_ is the fixed volume power density due to free convection cooling [W/m^3^] and r is the radial distance from the center of the body [m]. The values of these parameters are listed in Table 1.

The system is formulated as a state-space (SS) model with a zero-disturbance matrix (D = 0), as shown in equations (14) and (15), respectively [27]. The more accurate the model, the better are the predictions.


(14) $$x_{m}(k+1)=A_{m}x_{m}(k)+B_{m}u(k)$$

(15) $$y(k)=C_{m}x_{m}(k)$$

In equations (14) and (15), $x_{m}(k)$ is the state vector; y(k) is the output; and $A_{m}$, $B_{m}$, $C_{m}$ are the state, input, and output matrices, respectively. In this work, equation (3) is condensed, as shown in equation (16).

(16) $$\frac{\partial T_{b,n}(t)}{\partial t}=-\alpha (T_{b}-T_{n})+\text{KaH}(t)+\text{KbI}(t)$$

where,

(17) $$\alpha =\frac{\rho_{b}C_{b}}{\rho_{n}C_{n}}\omega_{b,n};\;K=\frac{h_{\text{free}}}{\rho_{n}C_{n}};\;a=0.5479\times 10^{5};b=7.8626\times 10^{5}$$

I(t) is an additional input introduced during the linearization process. The SS structures in equations (14) and (15) can then be formulated as

(18) $$\;A_{m}=[1-\alpha t_{s}];B_{m}=[{\text{Kat}}_{s}{\text{Kbt}}_{s}]\;\;C_{m}[1];D_{m}=[00]\;x_{m}(k)=[(T_{b}-T_{n})(k)];u(k)=\left[\begin{array}{c} H(k)\\ I(k) \end{array}\right]$$

The sampling time is represented by $t_{s}$, and I(k) does not represent a physical input; therefore, its amplitude is later constrained to a unit value. The states of the system are changed to state increments, and the output is included as one of the states. Then, equations (14) and (15) are transformed into an augmented model described by equations (19) and (20) [27].

(19) $$\left[\begin{array}{c} \Delta x_{m}(k+1)\\ y(k+1) \end{array}\right]=\left[\begin{array}{cc} A_{m} & 0_{m}^{T}\\ C_{m}A_{m} & I_{m} \end{array}\right]\left[\begin{array}{c} \Delta x_{m}(k)\\ y(k) \end{array}\right]+\left[\begin{array}{c} B_{m}\\ C_{m}B_{m} \end{array}\right]\Delta u(k)$$

(20) $$y(k)=[0_{m}\;\;I_{m}]\left[\begin{array}{c} \Delta x_{m}(k)\\ y(k) \end{array}\right]$$

where $0_{m}^{T}$ is a zero matrix and $I_{m}$ is an identity matrix.


(21) $$0_{m}^{T}=0;I_{m}=1$$

**Figure 2.**
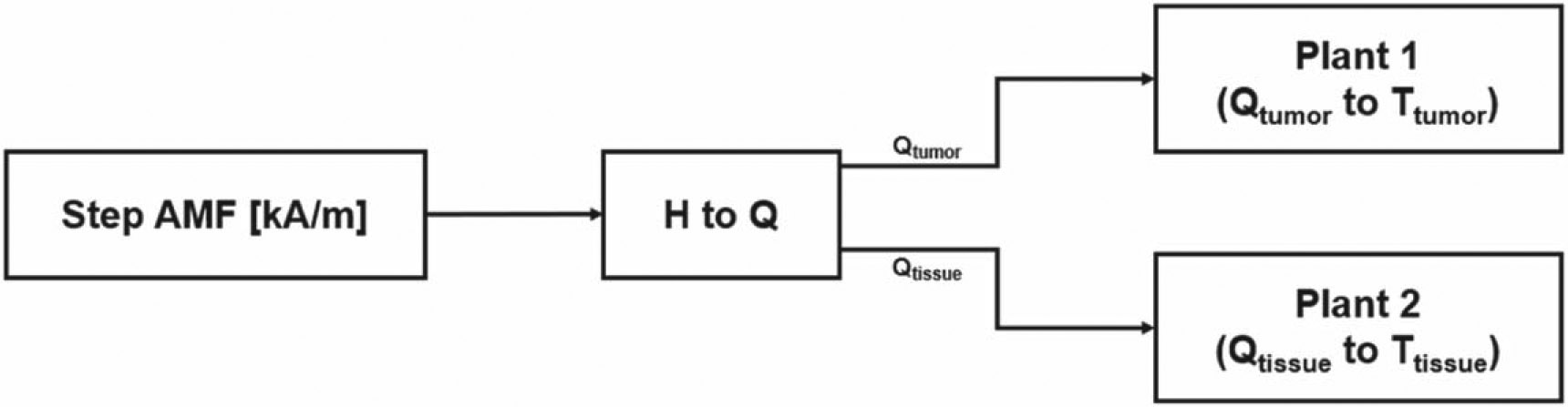
Block diagram of the simulation model representing the SS plant for tumor and healthy tissues.

Figure 2 shows a block diagram of the simulation model representing the SS plant for tumor and healthy tissues. The input to this model is the AMF signals.

**Figure 3.**
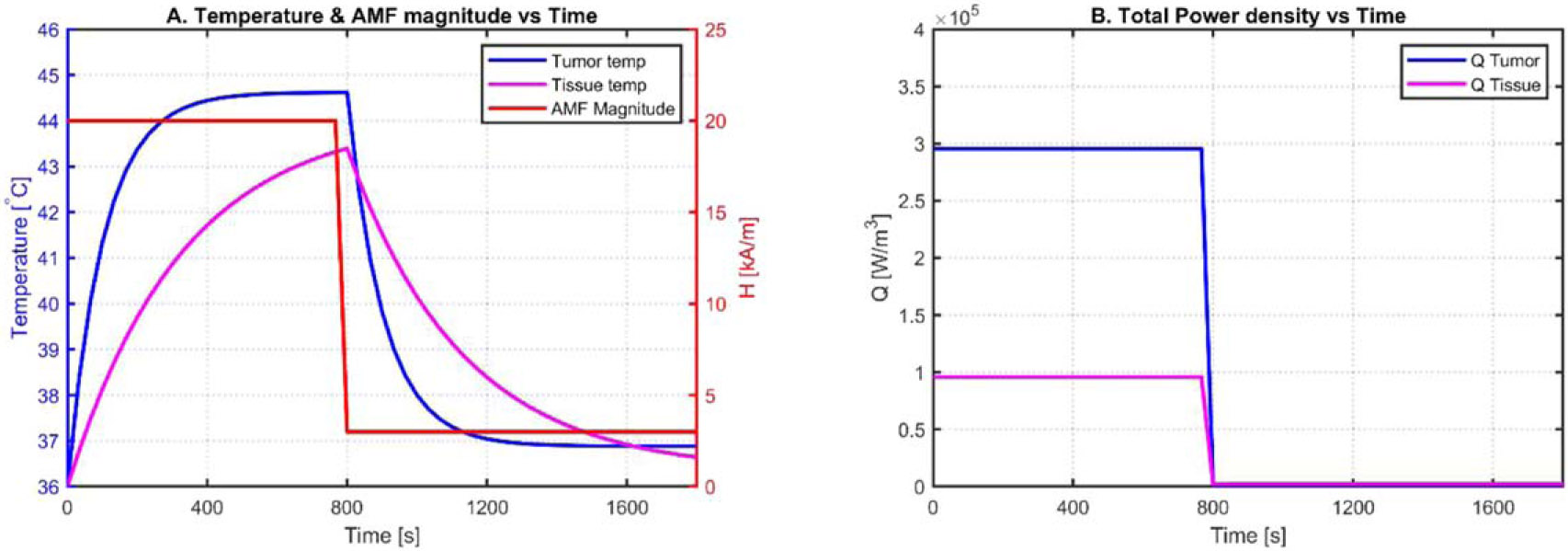
Tumor and healthy tissues LTI model simulation results.

The AMF magnitude, in figure 3(A), changes from a fixed value of 20 [kA/m] to 3 [kA/m] at 800 [s]; the volume power density shown in figure 3(B) changes accordingly from ~ 3 × 10^5^ [W/m^3^] and ~ 1 × 10^5^ [W/m^3^] to 0 [W/m^3^] at 800 [s], and the temperature response of the tumor and healthy tissue increased to ~ 44 [ °C] and ~ 43 [ °C], respectively, starting from a blood temperature of 36 [°C]. The MPC technique in this work aims to maintain a temperature of 43 [°C] at r = 1.0 [ cm], where the edge of the tumor is located, while maintaining a regulated temperature of 39 [°C] at r = 14.5 [cm], where the non-specific maximum temperature occurs in the healthy tissue. Considering this, the linear models expressed in equations (3) and (4) were simulated. The open-loop test results of the simplified tumor linear time invariant (LTI) model are shown in figure 3. These open-loop test results are used to design the MPC.

It should be noted that the rate of change of temperature in the tumor is faster than that in the healthy tissues, whereas the damping coefficient $\omega_{b,1}$, blood-perfusion [1/s] in the tumor, is greater than that in the tissue if $\rho_{n}C_{n}$ and $\rho_{b}C_{b}$ are fixed. Since a linear model is required to design the MPC, constant blood perfusion is modeled for two different domains, i.e., one for the healthy tissue, and one for the tumor, during the MPC design. This model is specified as Linear Time Invariant model. However, the blood perfusion changes with thermal dose. To account for this, temperature dependent blood perfusion was modeled for the plant, which was simulated using the MPC, as shown in the equation (23) [28, 29].

(22) $$D_{s}=1-e^{-A\int_{0}^{t}e^{-E_{a}∕\text{RT}(t)}\text{dt}}$$

(23) Ωb,n={ωb,n(30DS+1),(DS≤0.02)ωb,n(−13DS+1.86),(0.02<DS≤0.08)ωb,n(−0.79DS+0.884),(0.08<DS≤0.97)ωb,n(−3.87DS+3.87),(0.97<DS≤1.0)}

where, $\Omega_{b,n}$ is the time-varying perfusion rate and $\omega_{b,n}$ is the constant perfusion rate. $D_{s}$ is the degree of vascular damage, A is the frequency factor [1/s], $E_{a}$ is the activation energy [J/mol], R is the universal gas constant [J/K-mol] and T is the tissue/tumor temperature [K]. Based on this time varying perfusion the value of α from equation (17) has been modified as follows.


(24) $$\alpha =\frac{\rho_{b}C_{b}}{\rho_{n}C_{n}}\Omega_{b,n}$$

### 2.1. Constrained proportional integral control

A proportional Integral (PI) controller was designed, tuned, and implemented for tumor and healthy tissues. The general transfer function of the PI controller is given as

(25) $$\text{PI}(s)=K_{p}+K_{i}∕s$$

Where s is the Laplace variable, $K_{p}$ is the proportional gain and $K_{i}$ is the integral gain. The power density volume is applied across the tumor, organs, and tissue, which is a function of the tumor radius and AMF magnitude. Therefore, the power density due to the MNP, $Q_{\text{MNP}}$, is a function of SLP that varies non-linearly with respect to H [30, 31].

**Figure 4.**
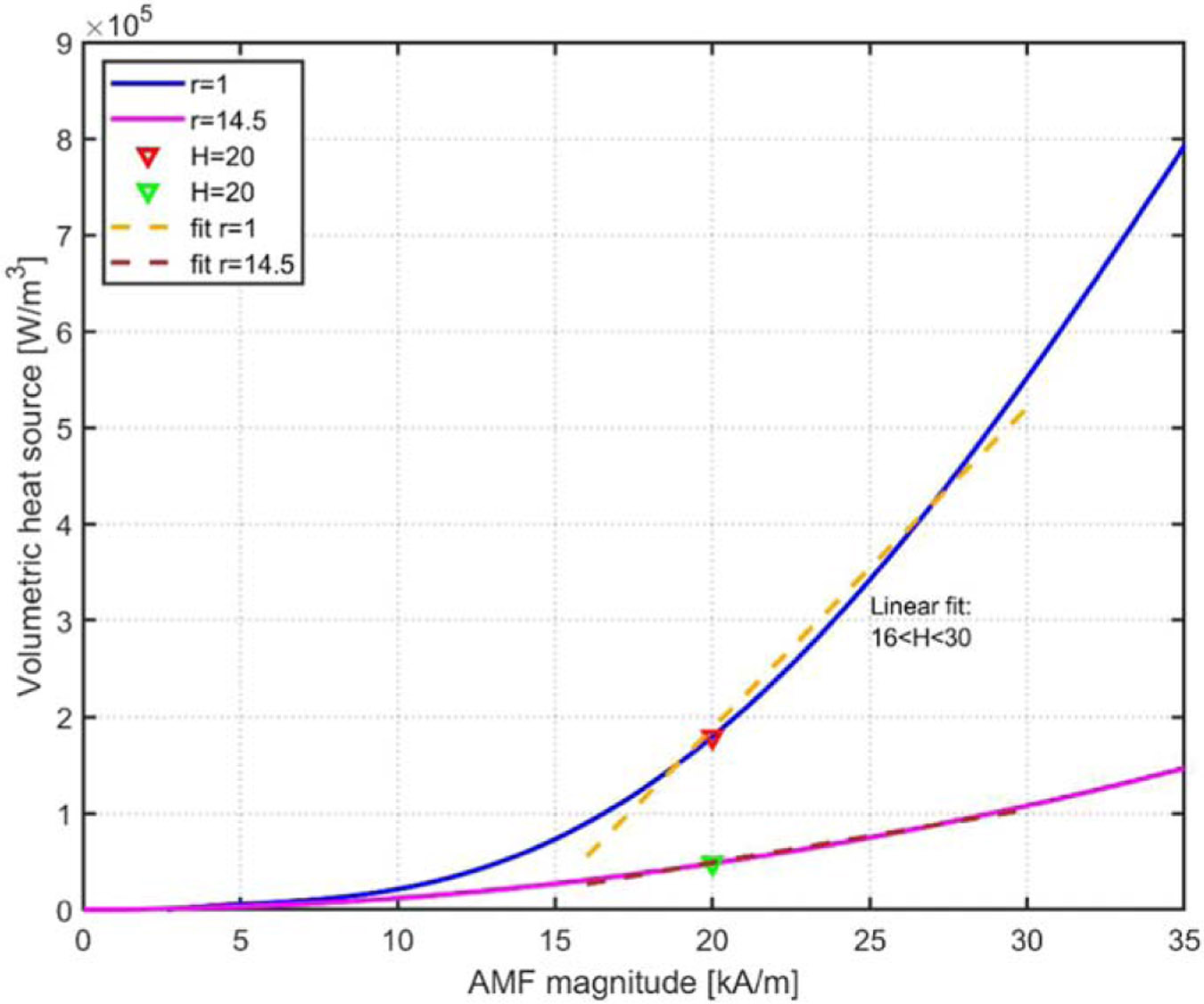
Linear power density distribution $Q_{\text{MNP}}+Q_{\text{eddy}}$ at different H.

The absolute root mean square error (RMSE) [32] and relative RMSE [33] were measured to ensure the accuracy of the linear fit of the H variable, as shown in figure 4.

**Figure 5.**
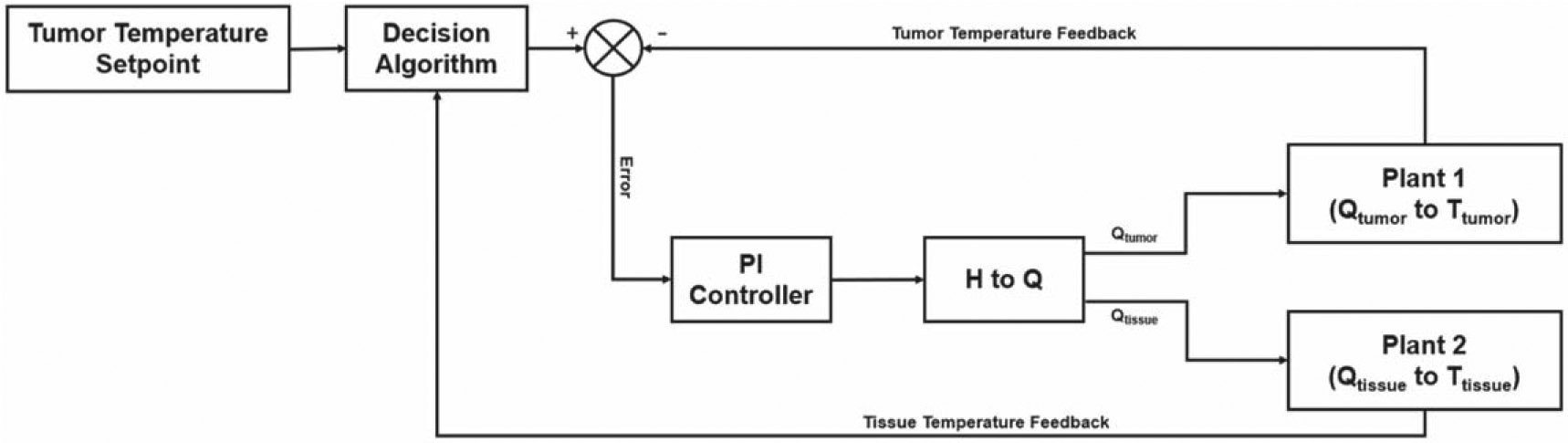
Constrained tumor temperature PI control with the nonlinear model.

The AMF produces undesired non-specific heating which increases quadratically with the radius from the body’s center, assuming that the body is precisely located at the center of the varying magnetic field. This secondary heating effect on the healthy tissue is taken into account in the design, as proposed in figure 5.

The decision algorithm works as follows:

- A tumor temperature setpoint of 43 [°C] is set at the tumor’s boundaries, letting the tumor temperature controller act as it would if there were no constraints.
- The tumor temperature rises faster than the healthy tissue temperature because the tumor has a higher perfusion rate; this means a lower time constant. As a result, the tumor temperature reaches a higher temperature than the healthy tissue in the same time period.
- Once the tumor temperature at its boundaries hits the upper limit of 39 [°C], a decision mechanism changes the tumor temperature setpoint back to the initial body temperature of 36 [°C].
- The tumor temperature control then tries to regulate the tumor temperature back to its initial state; consequently, the healthy tissue temperature decreases too.
- Provided that the tumor temperature has a lower perfusion rate at higher temperatures than the healthy tissue, the former decreases slower than the latter.
- After the healthy tissue temperature reaches a lower temperature limit of 38 [°C], the tumor temperature setpoint is changed back to its former value of 43 [°C], heating the tumor, and as a consequence of the AMF heating the healthy tissue too.

The time-varying blood perfusion term affects the tumor, making it heat faster but cool slower than the healthy tissue when the time increases. The nonlinear power input is a function of the AMF magnitude H, as follows:

(26) $$Q_{\text{in}1}(t)=Q_{m,1}+Q_{\text{lin}1}(t)$$

(27) $$Q_{\text{in}1}(t)=Q_{m,1}+1\times 10^{5}(0.5479H-7.8626)$$

(28) $$Q_{\text{in}1}(t)=0.5479H\times 10^{5}(t)-7.8626\times 10^{5}+Q_{m,1}$$

**Figure 6.**
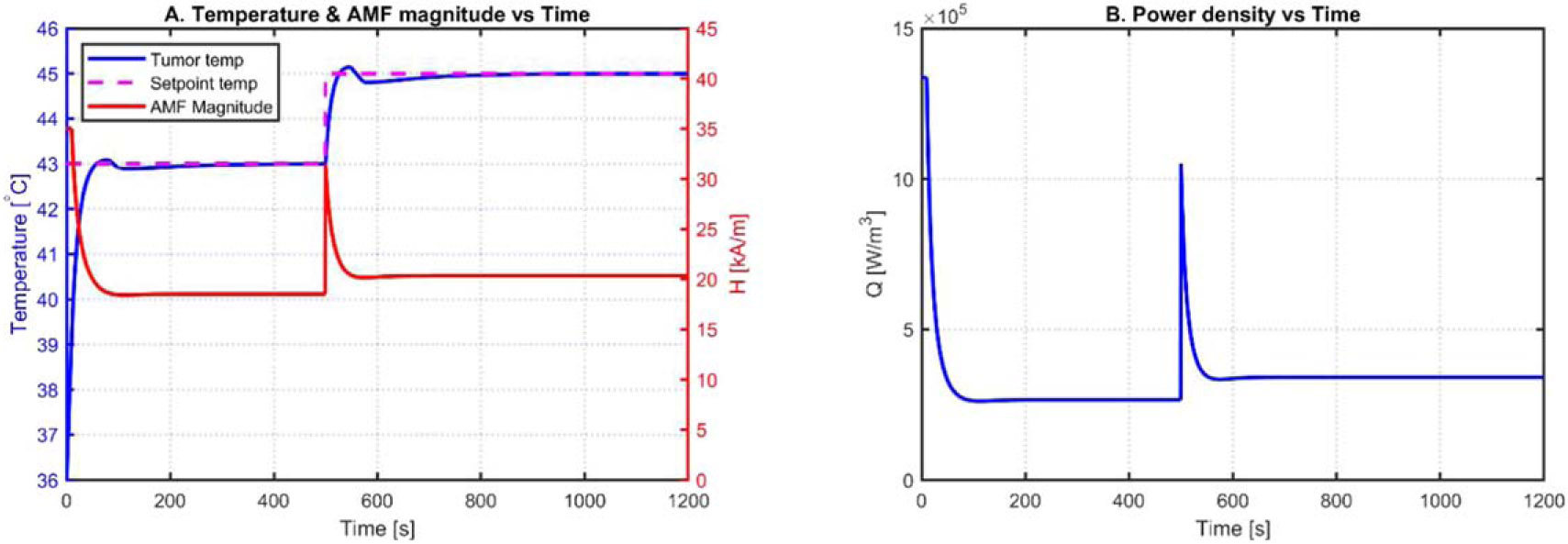
Constrained tumor temperature PI control response using Ziegler-Nicholas tuning method.

$Q_{m,1}$ is a time invariant term. The interaction between the nonlinear volume power density Q calculation and controller output H creates a slight variation in the steady-state tumor temperature, but it is corrected by the integral action of the PI controller. The closed-loop response of the PI controller using the nonlinear model is illustrated in figure 6. The PI control parameters $K_{p}$ and $K_{i}$ were tuned using the Ziegler-Nicholas method [34]. Figure 6(A) shows the variations in temperature and AMF magnitude with respect to time. Figure 6(B) shows the changes in power density with respect to time using the Zeigler-Nichols PI tuning method.

### 2.2. Unconstrained model predictive control

After the plant is modeled, a prediction horizon $N_{p}$ sample is established to make future predictions of the plant response subject to the control horizon $N_{c}$ and number of inputs based on the system’s initial state and the desired setpoint. The selection of $N_{p}$ and $N_{c}$ is very important because they provide more accurate information about the real response of the system and constitute the initial state for the new prediction window [24]. When $N_{p}$ predictions are shifted forward in time after a set of inputs, this is known as a moving horizon window. A special case called receding horizon control (RHC) is a strategy that executes the first element of an input trajectory to minimize the performance index at each time point [35]. The capacity of RHC to accommodate input/state restrictions is a significant advantage.

**Figure 7.**
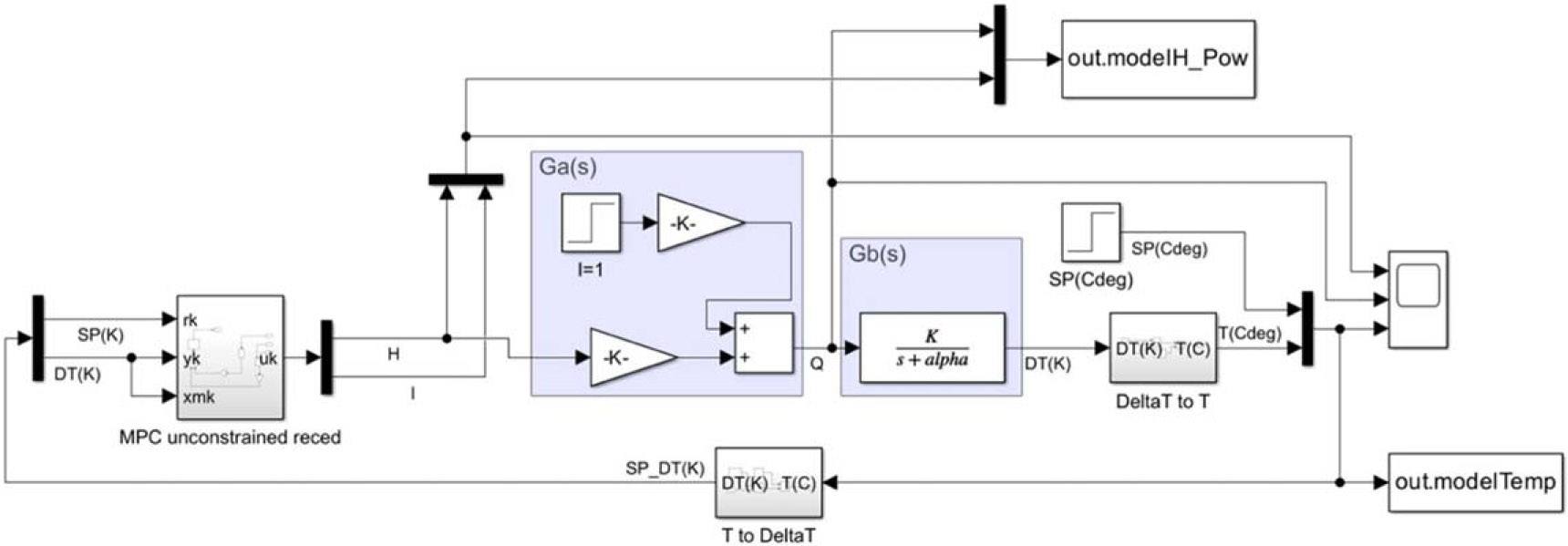
Unconstrained MPC model of the tumor and healthy tissues model.

Figure 7 shows the block diagram of unconstrained MPC for the tumor and healthy tissues. The MPC objective criterion minimizes the error between the actual output and the desired output or setpoint, giving importance to the input magnitude [35]. Block Ga(s) represents the transfer function in Laplace domain due to two inputs, i.e., the amplitude H and unit step input I to interpret the displacement of the linear approximation, as shown in equations (26) and (27).


(29) $$G_{a1}=\frac{Q_{\text{in}1}(s)}{H(s)}$$

(30) $$G_{a2}=\frac{Q_{\text{in}1}(s)}{I(s)}$$

Block Gb(s) interprets the dynamics for a volume power density input and a relative tumor temperature output response given by equation (28).


(31) $$G_{b}(s)=\frac{\left(T_{b}-T_{1})(s\right)}{Q_{\text{in}1}(s)}$$

The optimal input H is connected to the input of the LTI plant Ga(s) followed by the connection in series to the Gb(s) plant; the second unit input, I, coming from the MPC algorithm, is depicted in the top side of the blue area containing Ga(s); the output of the plant Gb(s) is followed by a transformation from relative temperature to absolute temperature. Lastly, a feedback-loop, containing the measured tumor temperature and setpoint, drives the variables of MPC block. Equation (29) defines the criterion or control objective as.

(32) $$J={\left(R_{s}-Y\right)}^{T}(R_{s}-Y)+\Delta U^{T}\bar{R}\Delta U$$

where Rs is a column vector of size $N_{p}$ containing the setpoint, $\bar{R}$ is a squared matrix containing the input weights, ΔU is the control path vector, and Y is the prediction vector of outputs based on the actual state and prediction horizon, and the last two are defined in equation (29).

(33) $$\;\Delta U={\left[\Delta u(k_{i})\;\;\Delta u(k_{i}+1)\;\cdots \;\Delta u(k_{i}+N_{c}-1)\right]}^{T}\;Y={\left[y(k_{i}+1\;|\;k_{i})\;\;y(k_{i}+2\;|\;k_{i})\;\cdots \;y(k_{i}+N_{p}\;|\;k_{i})\right]}^{T}$$

and Y can be calculated as

(34) $$Y=\text{Fx}(k_{i})+\Phi \Delta U$$

where $x(k_{i})$ is a vector with the current augmented state values, and F and Φ (Toeplitz) are matrices defined in equation (32).


(35) $$\;F={\left[\text{CA}\;\;{\text{CA}}^{2}\;\;{\text{CA}}^{3}\;\cdots \;{\text{CA}}^{N_{p}}\right]}^{T}\;\Phi =\left[\begin{array}{cccc} \text{CB} & 0 & \cdots & 0\\ \text{CAB} & \text{CB} & \cdots & 0\\ \vdots & \vdots & \ddots & \vdots \\ {\text{CA}}^{N_{p}-1}B & {\text{CA}}^{N_{p}-2}B & \cdots & {\text{CA}}^{N_{p}-N_{c}}B \end{array}\right]$$

**Figure 8.**
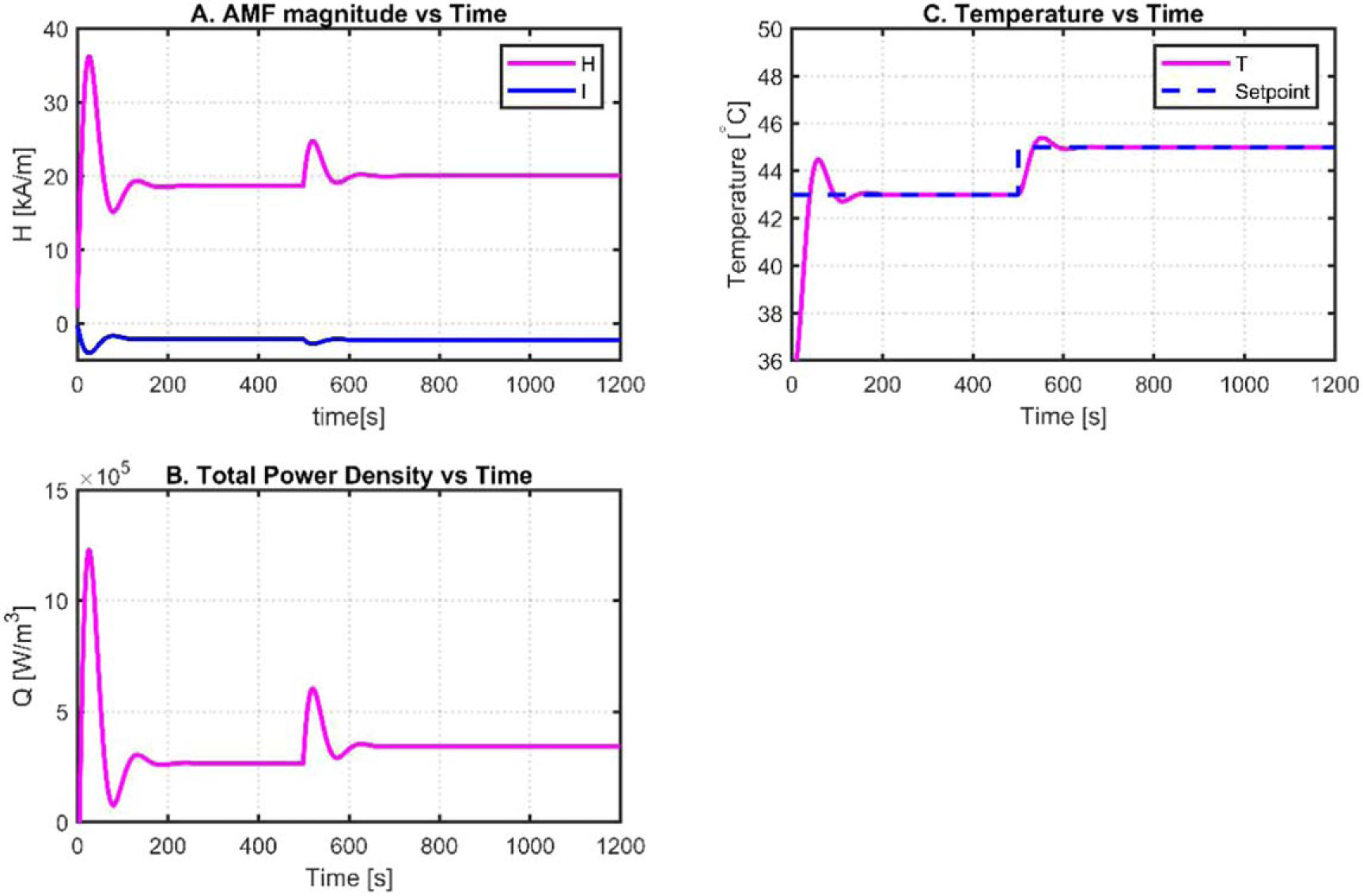
Unconstrained MPC simulation results of the tumor and healthy tissues.

The ${\left(R_{s}-Y\right)}^{T}(R_{s}-Y)$ term in equation (29) aims to minimize the squared error between the output and setpoint, whereas the term $\Delta U^{T}\bar{R}\Delta U$ emphasizes how large the input can be. The input that minimizes equation (29) is

(36) $$\Delta U={\left(\Phi^{T}\Phi +\bar{R}\right)}^{-1}\Phi^{T}(R_{s}-\text{Fx}(k_{i}))$$

where the column vector containing the setpoint values, $R_{s}$, is divided into a unit vector, ${\bar{R}}_{s}={\left[1\;\;1\;\cdots \;1\right]}^{T}$, multiplied by the scalar initial setpoint $r(k_{i})$, that is, $R_{s}={\bar{R}}_{s}r(k_{i})$, and $x(k_{i})$ is a column vector with the initial augmented states. The results for unconstrained MPC are shown in figure 8.

### 2.3. Constrained model predictive control

In the MPC design, the saturation approach was tested, and despite providing working results, the behavior of the MPC was no longer optimal. Instead, in this section, the input constraints are included as a set of inequalities that must be computed, along with the cost function of equation (29), which guarantees optimal constrained control. Because the constraints are only on the plant inputs, the cost function J, is expressed as a function of the input increment ΔU by substituting equation (26) into equation (24) to obtain equation (27).


(37) $$J={\left(R_{s}-\text{Fx}(k_{i})\right)}^{T}(R_{s}-\text{Fx}(k_{i}))-2\Delta U^{T}\Phi^{T}(R_{s}-\text{Fx}(k_{i}))+\Delta U^{T}(\Phi^{T}\Phi +\bar{R})\Delta U$$

**Figure 9.**
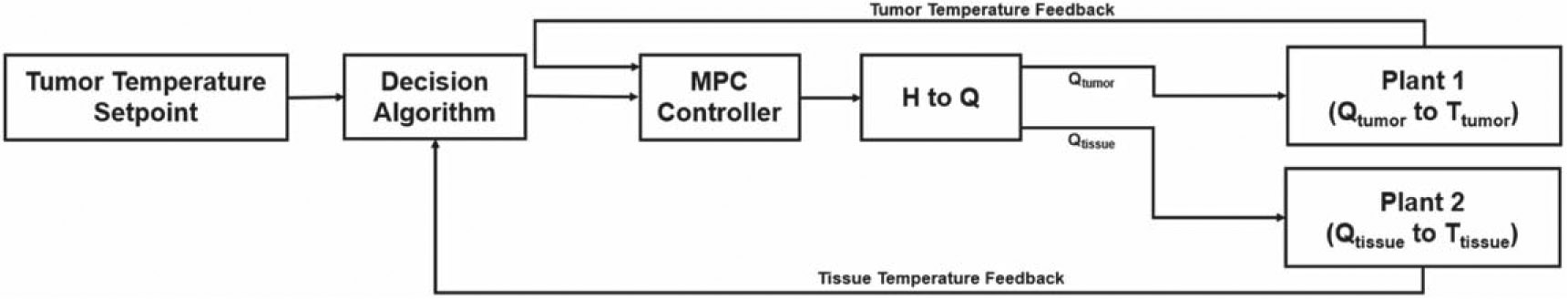
Constrained MPC model of the tumor and healthy tissues model.

Hildreth’s Quadratic Algorithm was used to minimize the cost function of equation (27), subject to constraints on the input vector ΔU [36]. This algorithm immediately obtains an unconstrained solution. Subsequently, a recursive technique is used to calculate the Lagrange multipliers Λ. matrix Λ provides the necessary changes to the unconstrained optimal solution $x_{h0}$ to generate the constrained optimal solution $x_{h}^{*}$. The difference in the values of the constrained solutions between steps is known as the error. The algorithm may be terminated if the error is less than the random convergence criterion. MPC, with a constraint on the AMF amplitude and increment, was used to control tumor temperature. An additional constraint was set on the healthy tissue temperature to operate below 39 [°C]. To add the latter constraint, the fixed tumor temperature setpoint of 43 [°C] was replaced with a hysteresis mechanism with a lower setpoint limit of 42 [°C] and an upper setpoint limit of 45 [°C]. Hysteresis boundaries were determined during the simulation to improve the performance of the MPC when it worked outside the linear region. The constrained MPC model was structured and implemented as shown in figure 9. The decision algorithm has a working principle similar to the one explained in PI controller.

Plant input H is applied to the tumor and tissue temperature model shown at the center of figure 9. The input attributes of the MPC controller are the relative temperature, relative temperature increment, and temperature reference, and the output attributes are the constrained optimal plant inputs H and I. These constraints are formulated as shown in equations (31) and (32), respectively:

(38) $$0\leq H\leq 36\;\left(\frac{\text{kA}}{m}\right),\text{and}-4\leq \Delta H\leq 10\left(\frac{\text{kA}}{m}\right)∕s$$

(39) $$0.95\leq I\leq 1.05\;\left(\frac{\text{kA}}{m}\right),\text{and}\;-0.5\leq \Delta I\leq 0.5\left(\frac{\text{kA}}{m}\right)∕s$$

**Figure 10.**
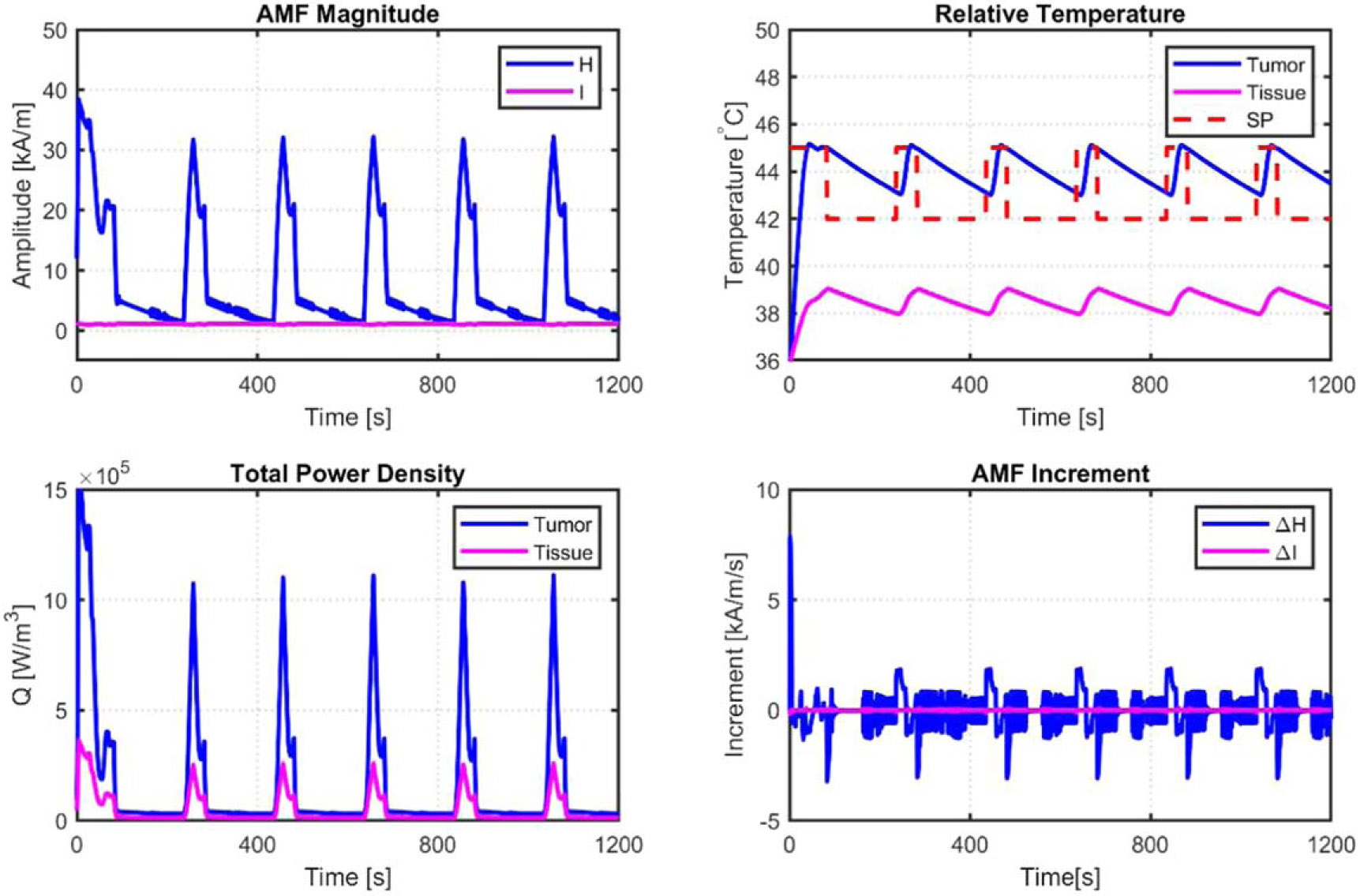
Constrained MPC simulation results of the tumor and healthy tissues nonlinear model.

The behavior of the AMF amplitude H, as shown in figure 10(A), resembles a distorted square signal with a very high peak at each rising edge. Emphasis is placed on the tumor and tissue temperature limits observed in figure 10(B), where it is seen that the tumor temperature oscillates between 43.5 [°C] and 45 [°C] and the tissue temperature between 38 [°C] and 39 [°C]; the tumor temperature setpoint (SP) changes to 42 [°C] when the tissue temperature reaches 39 [°C] and changes back to 45 [°C] when the tissue temperature is safely maintained at 38 [°C]. The inputs’ H and I (secondary input introduced because of the linearization) amplitudes are shown in figure 8(A), and their rate of change is shown in figure 10(D). The total volume power density applied to the tumor and healthy tissues is shown in figure 10(C). The healthy tissue temperature is a safety constraint for the treatment and from medical device point of view, this becomes the safety stopping criteria for the controller. Hence, this constraint is imposed outside the MPC, so that in any case, if the controller fails, this becomes the safety trigger.

## 3. Results

**Figure 11.**
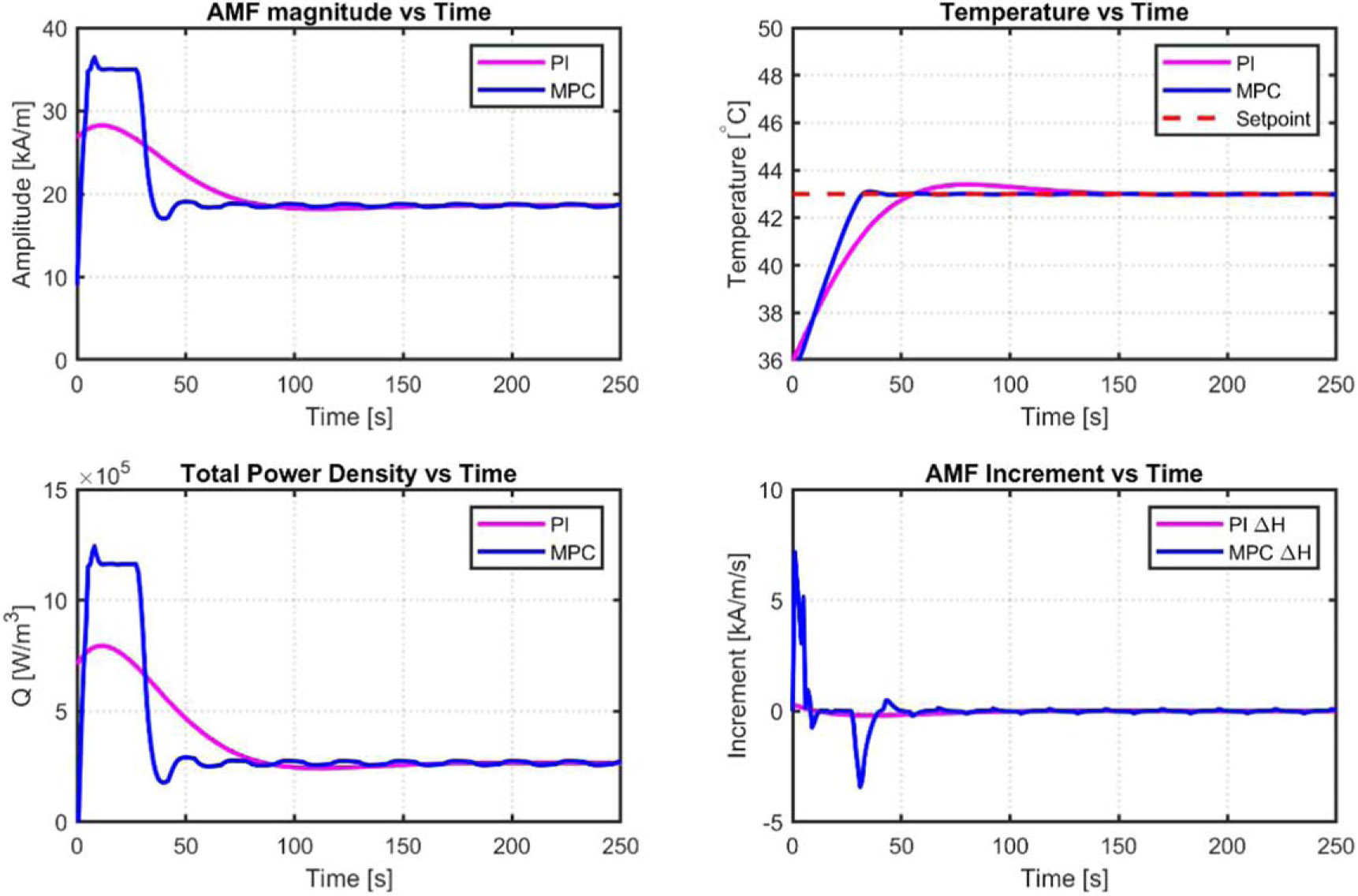
Constrained MPC model simulation results of the tumor and healthy tissues.

A comparison between the PI and MPC control methods is shown in figure 11. The MPC response reaches the setpoints of the tumor and tissue temperatures faster than the PI approach. A summary of the performance criteria, including settling time, peak time, overshoot percentage, maximum and minimum control increment, and maximum control output, is listed in table 2.

**Figure 12.**
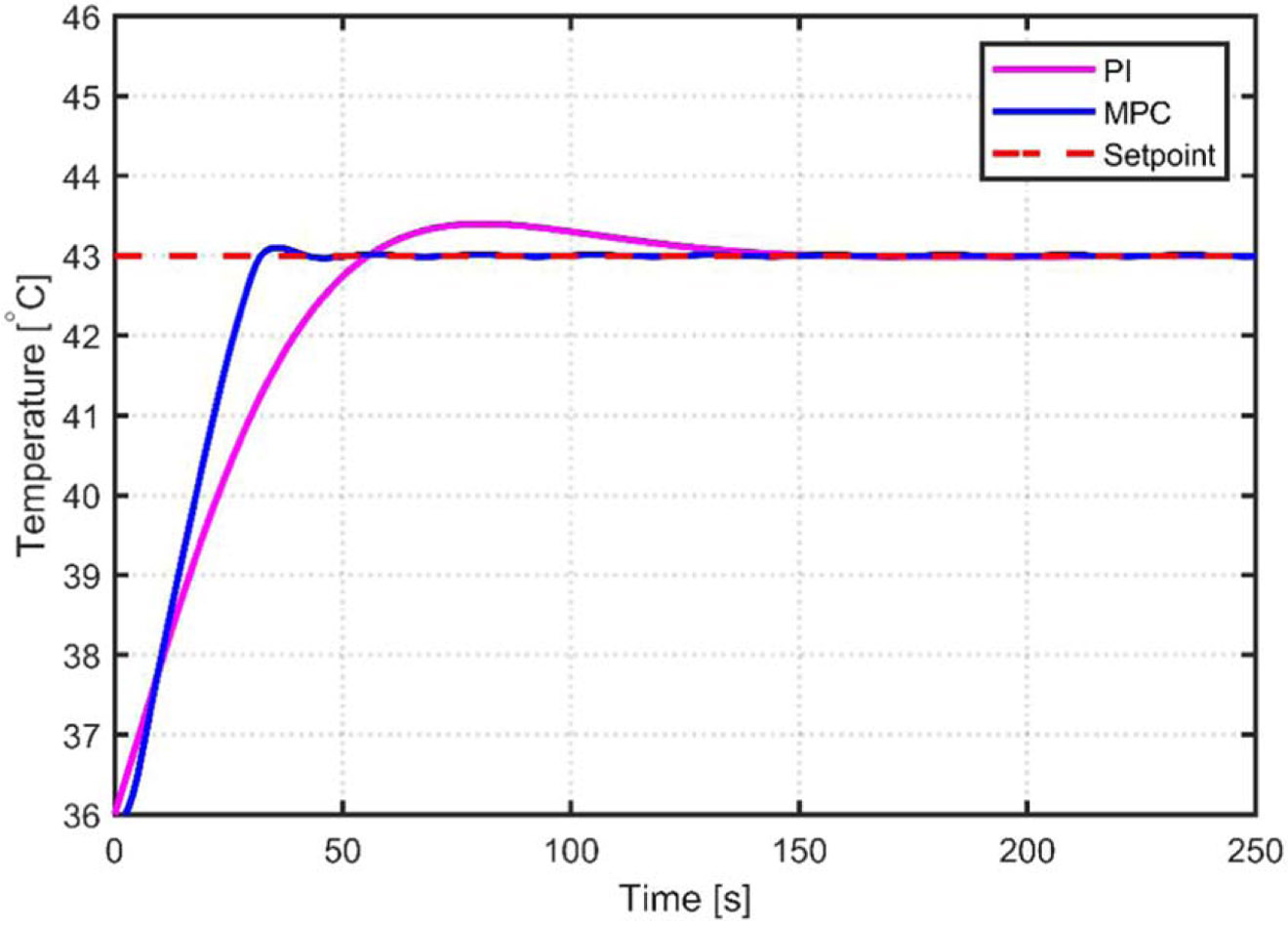
Constrained MPC versus PI simulation results for tumor and healthy tissues.

A clearer comparison of the constrained MPC versus PI results can be seen in figure 12. The minor oscillations for the constrained MPC can be seen.

## 4. Discussion

General problems associated with MNPH treatment include generation of eddy current heating when exposed to radiofrequency AMF. An effective MNPH treatment can be achieved when the hysteresis loss deposited by the iron-oxide nanoparticles is significantly greater than the eddy current heating. Pulse width modulation can be used to reduce the eddy current effect; however, even with this approach, it is difficult to maintain the temperatures of the surrounding healthy tissues below the necrosis temperature. MPC can be applied to MNPH treatment to control tumor temperatures below the necrosis temperature and overcome this limitation [18, 19].

In this study, the MPC algorithm was applied to MNPH to regulate the temperature in the range 42 [°C] to 45 [°C]. It was determined that this nature was an oscillatory temperature profile at the beginning, followed by a stable trend. Additionally, the simulation results suggest that no less than 400 [s] is required for the oscillations to end and for the tumor temperature at the edge to reach a steady state. The explanation for the oscillations is that the temperature response in the tumor is initially faster than that in the healthy tissue. As more thermal damage occurs in the tumor and the perfusion rate decays from its initial value to zero, the temperature response in the tumor begins to slow down. This change in tumor dynamics requires a less intense heat source to maintain hyperthermia, making it easier for the controller to maintain a temperature of up to 43 [°C]. Moreover, the MPC algorithm design was modified to include a recursive calculation of Φ and F matrices according to the plant changes in the tumor model owing to the time-varying perfusion rate. Recall that matrices Φ and F are fixed when the plant is an LTI. This variation in the MPC algorithm can be considered as an adaptive approach.

As an example of the limitations of this study, the point at the center of the tumor was considered to have the highest temperature, and it was considered the only source of heat transfer to the edge of the tumor. Another challenge of this research was to model a plant that represents temperature changes due to external heat sources and heat transfer. The treatment plan cannot be directly translated into a clinical setting because the model used in this approach is a simplified one-dimensional model. In addition, the control parameters for the MPC must be designed according to the complexity of the model. The model is linearized with assumptions and fits and has the drawback of unpredicting nonlinear responses to the changes in tissue properties when exposed to high temperatures. After performing verification studies, it is important to validate the model with experimental settings for translation into the clinic according to the American Society of Mechanical Engineers standards to verify and validate simulation outcomes (ASMEV-VUQ40) [37, 38].

Our future studies will focus on implementing the MPC strategy in a three-dimensional model for a more accurate representation of temperature control [39]. The controller stability will be verified and validated with experimental settings according to the ASMEV-VUQ40 standards for the treatment plan to be translated into the clinic [37, 38].

## 5. Conclusions

The MPC methodology was designed to control tumor temperature by considering the effect of eddy current heating. One constraint on the main input signal H and another constraint on the increment ΔH are applied to the MPC controller. The simulation results show the likelihood of successful MPC implementation over the MNPH treatment. More importantly, the MPC method showed significant advantages over the PI controller in handling constraints in the main input of the hyperthermia treatment process. In future work, a more precise time-varying blood perfusion rate will be considered in the modeling process to improve controller performance.

## Figures and Tables

**Table 1.** Free convection parameters.

Property	Value
$h_{\text{free}}$ [W/m^2^-K]	50
r[m]	14.5 × 10^−2^
$Q_{\text{free}}$ [W/m^3^]	4.83 × 10^3^

**Table 2.** Time-domain behavior comparison between PI and MPC over the LTI tumor temperature response.

Parameter	Controllers	
	PI	MPC
Tumor temperature peak time [s]	40	26
Tumor temperature settling time [s]	115	30
Tumor temperature overshoot [%]	5.7	1.4
Maximum temperature control output H [kA/m]	28.3	36
Minimum temperature control output H [kA/m]	18.8	9.0
Maximum control increment ΔH [kA/m/s]	0.3	7.2
Minimum control increment ΔH [kA/m/s]	−0.2	−3.5

## Data Availability

All data that support the findings of this study are included within the article (and any supplementary files).

## References

[1] Hasselbalch B, Lassen U, Hansen S, Holmberg M, Sorensen M, Kosteljanetz M and Poulsen HS 2010 Cetuximab, bevacizumab, and irinotecan for patients with primary glioblastoma and progression after radiation therapy and temozolomide: a phase II trial Neuro-oncology 12 508–16 20406901 10.1093/neuonc/nop063 PMC2940618

[2] Clarke J, Butowski N and Chang S 2010 Recent advances in therapy for glioblastoma Arch. Neurol 67 279–83 20212224 10.1001/archneurol.2010.5

[3] Kashevsky BE, Kashevsky SB and Prokhorov IV 2009 Dynamic magnetic hysteresis in a liquid suspension of acicular maghemite particles Particuology 7 451–8

[4] Oleson JR 1982 Hyperthermia by magnetic induction: I. Physical characteristics of the technique International Journal of Radiation Oncology 8 1747–56 10.1016/0360-3016(82)90297-8 7153086

[5] Dennis CL and Ivkov R 2013 Physics of heat generation using magnetic nanoparticles for hyperthermia Int. J. Hyperth 29 715–29 10.3109/02656736.2013.836758 24131317

[6] Perrin J 1908 Molecular agitation and the brownian movement CRAS 146 967–70

[7] Branquinho LC, Carrião MS, Costa AS, Zufelato N, Sousa MH, Miotto R and Bakuzis AF 2013 Effect of magnetic dipolar interactions on nanoparticle heating efficiency: implications for cancer hyperthermia Sci. Rep 3 2887 24096272 10.1038/srep02887 PMC3791447

[8] Das R, Langou S, Le TT, Prasad P, Lin F and Nguyen TD 2022 Electrical stimulation for immune modulation in cancer treatments Front. Bioeng. Biotechnol 9 795300 35087799 10.3389/fbioe.2021.795300 PMC8788921

[9] Attaluri A, Kandala SK, Zhou H, Wabler M, DeWeese TL and Ivkov R 2020 Magnetic nanoparticle hyperthermia for treating locally advanced unresectable and borderline resectable pancreatic cancers: the role of tumor size and eddy-current heating Int. J. Hyperth 37 108–19 10.1080/02656736.2020.1798514 PMC8363047 33426990

[10] Tofighi MR and Attaluri A 2021 Closed-loop pulse-width modulation microwave heating with infrared temperature control for perfusion measurement IEEE Trans. Instrum. Meas 70 1–733776080

[11] Ahmed A, Kim E, Jeon S, Kim J and Choi H 2022 Closed-loop temperature-controlled magnetic hyperthermia therapy with magnetic guidance of superparamagnetic iron-oxide nanoparticles 5 2100237

[12] Du Z, Sun Y, Liu J, Su R, Yang M, Li N, Gan Y and Ye N 2018 Design of a temperature measurement and feedback control system based on an improved magnetic nanoparticle thermometer Meas. Sci. Technol 29 045003

[13] Sung S and Lee B 1996 Limitations and countermeasures of PID controllers Journal of Industrial Engineering and Chemistry Research 35 2596–610

[14] VanBaren P and Ebbini ES 1995 Multipoint temperature control during hyperthermia treatments: theory and simulation IEEE Trans. Biomed. Eng 42 818–27 7642196 10.1109/10.398643

[15] Abu-Ayyad M, Dubay R and Hernandez J 2009 Application of infinite model predictive control methodology to other advanced controllers ISA Trans. 48 54–61 18778821 10.1016/j.isatra.2008.07.007

[16] Abu-Ayyad M, Abu-Mahfouz I and Banerjee A 2013 Conventional and predictive control algorithms for controlling nonlinear processes using multiple-model approach Journal of Mechanics Engineering and Automation 3 22–28

[17] Sebeke L, Deenen D, Maljaars E, Heijman E, de Jager B, Heemels W and Grüll H 2019 Model predictive control for MR-HIFU-mediated, uniform hyperthermia Int. J. Hyperth 36 1039–49 10.1080/02656736.2019.1668065 31621435

[18] Arora D, Skliar M and Roemer RB 2002 Model-predictive control of hyperthermia treatments IEEE Trans. Biomed. Eng 49 629–39 12083297 10.1109/TBME.2002.1010846

[19] Arora D, Skliar M and Roemer RB 2005 Minimum-time thermal dose control of thermal therapies IEEE Trans. Biomed. Eng 52 191–200 15709656 10.1109/TBME.2004.840471 PMC3703959

[20] Pennes H 1948 Analysis of tissue and arterial blood temperatures in the resting human forearm 1 93–122 10.1152/jappl.1948.1.2.93 18887578

[21] Deenen D, Maljaars E, Sebeke L, de Jager B, Heijman E, Grüll H and Heemels W 2018 Offset-free model predictive control for enhancing MR-HIFU hyperthermia in cancer treatment IFAC-PapersOnLine 51 191–6

[22] van Wordragen J 2019 Hierarchical mixed-integer model predictive control for real-time large-area MR-HIFU hyperthermia PhD Thesis, Master’s thesis Eindhoven University of Technology

[23] Huibregtse R, Deenen D, Nouwens S, Heemels W, Paulides M and de Jager A 2020 Model predictive control for magnetic-resonance-guided radiofrequency hyperthermia Phd Thesis Mechanical Engineering Department

[24] Attaluri A, Jackowski J, Sharma A, Kandala SK, Nemkov V, Yakey C, DeWeese TL, Kumar A, Goldstein RC and Ivkov R 2020 Design and construction of a maxwell-type induction coil for magnetic nanoparticle hyperthermia Int. J. Hyperth 37 1–14 10.1080/02656736.2019.1704448 PMC6956739 31918595

[25] Salmon D 2001 Thermal conductivity of insulations using guarded hot plates, including recent developments and sources of reference materials Meas. Sci. Technol 12 R89

[26] Soetaert F, Dupre L, Ivkov R and Crevecoeur G 2015 Computational Evaluation of Amplitude Modulation for Enhanced Magnetic Nanoparticle Hyperthermia Biomedical Engineering/Biomedizinische Technik 60 491–504 26351900 10.1515/bmt-2015-0046

[27] Wang L 2009 Model predictive control system design and implementation using MATLAB (Springer) 3

[28] He X, McGee S, Coad JE, Schmidlin F, Iaizzo PA, Swanlund DJ and Bischof JC 2004 Investigation of the thermal and tissue injury behaviour in microwave thermal therapy using a porcine kidney model Int. J. Hyperth 20 567–93 10.1080/0265673042000209770 15370815

[29] Schutt DJ and Haemmerich D 2008 Effects of variation in perfusion rates and of perfusion models in computational models of radio frequency tumor ablation Med. Phys 35 3462–70 18777906 10.1118/1.2948388 PMC2673648

[30] Bekovic M and Hamler A 2010 Determination of the heating effect of magnetic fluid in alternating magnetic field IEEE Trans. Magn 46 552–5

[31] Garaio E, Sandre O, Collantes J, Garcia J, Mornet S and Plazaola F 2015 Specific absorption rate dependence on temperature in magnetic field hyperthermia measured by dynamics hysteresis losses (ac magnetometry) Nanotechnology 26 015704 25490677 10.1088/0957-4484/26/1/015704

[32] Chai T and Draxler R 2014 Root mean square error (RMSE) or mean absolute error (MAE)?—arguments against avoiding RMSE in the literature Geoscientific Model Development 7 1247–50

[33] Willmott C and Matsuura K 2005 Advantages of the Mean Absolute Error (MAE) over the Root Mean Square Error (RMSE) in assessing average model performance Climate Research 30 79–82

[34] Åström K and Hägglund T 2004 Revisiting the Ziegler-Nicholas step response method for PID control J. Process Control 14 635–50

[35] Lee Y, Kouvaritakis B and Cannon M 2002 Constrained receding horizon predictive control for nonlinear systems Automatica 38.12 2093–102

[36] Iusem AN and De Pierro AR 1990 On the convergence properties of Hildreth’s quadratic programming algorithm Math. Program 47 37–51

[37] Bischoff J 2023 Asme VVUQ 40 verification, validation, and uncertainty quantification in computational modeling of medical devices Available online: (https://players.brightcove.net/1711318824001/default_default/index.html?videoId=6076214051001) (accessed on 29, 2023)

[38] Kandala SK, Sharma A, Mirpour S, Liapi E, Ivkov R and Attaluri A 2021 Validation of a coupled electromagnetic and thermal model for estimating temperatures during magnetic nanoparticle hyperthermia Int. J. Hyperth 38 611–22 10.1080/02656736.2021.1913244 PMC8363028 33853493

[39] Lad Y, Jangam A, Carlton H, Abu-Ayyad M, Hadjiapanayis C, Ivkov R, Zacharia B and Attaluri A 2023 Development of a Treatment Planning Framework for Laser Interstitial Thermal Therapy (LITT) Cancers 15 4554–65 37760524 10.3390/cancers15184554 PMC10526178

